# High throughput isolation of RNA from single-cells within an intact tissue for spatial and temporal sequencing a reality

**DOI:** 10.1371/journal.pone.0289279

**Published:** 2023-08-01

**Authors:** John Stanley, Akshar Lohith, Lucca Debiaso, Kevan Wang, Minh Ton, Wenwu Cui, Weiwei Gu, Aihua Fu, Nader Pourmand

**Affiliations:** 1 Department of Biomolecular Engineering, University of California, Santa Cruz, California, United States of America; 2 NVIGEN Inc, Campbell, California, United States of America; Teikyo University, School of Medicine, JAPAN

## Abstract

Single-cell transcriptomics is essential for understanding biological variability among cells in a heterogenous population. Acquiring high-quality single-cell sequencing data from a tissue sample has multiple challenges including isolation of individual cells as well as amplification of the genetic material. Commercially available techniques require the isolation of individual cells from a tissue through extensive manual manipulation before single cell sequence data can be acquired. However, since cells within a tissue have different dissociation constants, enzymatic and mechanical manipulation do not guarantee the isolation of a homogenous population of cells. To overcome this drawback, in this research we have developed a revolutionary approach that utilizes a fully automated nanopipette technology in combination with magnetic nanoparticles to obtain high quality sequencing reads from individual cells within an intact tissue thereby eliminating the need for manual manipulation and single cell isolation. With the proposed technology, it is possible to sample an individual cell within the tissue multiple times to obtain longitudinal information. Single-cell RNAseq was achieved by aspirating only1-5% of sub-single-cell RNA content from individual cells within fresh frozen tissue samples. As a proof of concept, aspiration was carried out from 22 cells within a breast cancer tissue slice using quartz nanopipettes. The mRNA from the aspirate was then selectively captured using magnetic nanoparticles. The RNAseq data from aspiration of 22 individual cells provided high alignment rates (80%) with 2 control tissue samples. The technology is exceptionally simple, quick and efficient as the entire cell targeting and aspiration process is fully automated.

## Introduction

Since the formulation of cell theory by Jakob Schleiden and Theodor Schwann [1] and further discovery by Rudolph Virchow that new cells are formed by cell division, and tissues by cell multiplication, development of technologies to interrogate biology in single cells had gained importance. Subsequently, the discovery of DNA led to the evolution of modern genetics and genomics and has been primarily adopted to study variations within an ecosystem or organism. However, genetic variations among cells within a population can provide valuable information not accessible through bulk approaches. In this regard, single-cell genomics have been pivotal in addressing important biological questions that are possible only through interrogating single cells. The path to acquiring single-cell genomic data is challenging as it requires the isolation of a single cell from a population followed by amplification to obtain sufficient genetic material for downstream analysis. It is also important for the technology to be cost-effective and identify the artifacts introduced from cell isolation and amplification [2].

Isolation of viable individual cells from a tissue is the critical first step and generally requires enzymes or mechanical force. It is well understood that different subset of cells within a tissue can have different dissociation constants. Hence during enzymatic or mechanical dissociation, it is possible that only cells from specific subsets are isolated and do not necessarily include all of the heterogenic population. To overcome this drawback, techniques such as laser-capture microdissection [3], microfluidic [4] and bead-based [4] methods, and fluorescence-activated cell sorting (FACS) have been extensively employed for isolating individual cells. But these techniques often suffer from low throughput, poor sequencing data, the need for extensive manual manipulation, and confirmation of single-cell isolation. In this regard, nanopipettes with pore diameters of less than 200 nm have proved to be an innovative tool for a wide array of single-cell applications including nanoinjection [5–7], nanoaspiration/nanobiopsy [8,9], and nanosensing—the measurement of biomolecules at subcellular levels [10–14] and electrochemical imaging of cell surfaces. A nanopipette based single-cell nanobiopsy platform can be used carry out intracellular sampling while maintaining cell viability, as detailed by *Actis et al*. [8]. Aspiration is achieved through voltage-controlled influx and can be used to precisely aspirate volumes as small as a few picolitres. Our group has extensively used nanopipettes for aspiration of RNA and has successfully performed Next-Generation Sequencing (NGS) from the genetic material extracted with nanopipettes [8]. Moreover, our group has also published on the technological development of a fully automated system that can very accurately insert a nanopipette into a target cell of interest and aspirate/inject a controlled volume of material without destroying the cell [8]. With the possibility of extracting minute volumes of material with a fully automated nanopipette, we can deploy the nanopipette platform to extract genetic material from multiple individual cells within the tissue.

Single-cell transcriptomics has enabled the study of cellular diversity within a heterogenous tissue. However, currently available commercial technologies fail to provide multiple measurements from a single cell, which is required for obtaining reliable data [15]. To overcome the need for multiple sampling and to preserve spatial information, in situ hybridization (ISH) or sequencing can be used to profile a transcriptome in fixed cells or tissues. While single molecule florescence in situ hybridization (smFISH) has been the been the go-to technique for single-cell transcriptomics [16,17], hybrid protocols using high resolution microscopy have been used to image large copies of mRNA [18,19]. Recent innovations such as sequential FISH (seqFISH) [20] and amplified seqFISH [21] for temporal barcoding have also been utilized. Nevertheless, smFISH based tools are plagued by high background in tissues making it a challenge to use with tissue samples. An alternate approach is to use tailor-made magnetic nanoparticles to capture the mRNA from very minute quantities of the sample. Magnetic nanoparticles have been successfully utilized for a wide variety of applications including cfDNA extraction [22], DNA size selection [23,24], nucleic acid capture [25,26], cell separation and exosome capture [27,28], protein binding, purification [29] and immunodiagnostics [30], immunoprecipitation [31,32] and *in vivo* imaging and nanoparticle drug delivery [33,34].

From a literature review it is evident that the progress of basic scientific research and the development of more effective therapeutics relies greatly on genomic studies of single cells within a tissue. Also apparent is that, isolating individual cells from a tissue and obtaining good sequence reads is challenging and time-consuming. Keeping these factors in mind, we have developed a novel approach using the nanopipette technology in combination with the magnetic nanoparticles from NVIGEN to obtain high-quality sequencing reads from individual cells within a tissue. As a proof of concept, aspiration was carried out from 22 cells within a breast cancer tissue slice using quartz nanopipettes. As described by *Actis et*. *al*. [8], the nanopipette was adapted as a Scanning Ion-Conductance Microscopy (SICM) platform to extract precise quantities of cytoplasmic material. Collaboration with Yokogawa Electric ensured that the SICM platform was fully automated and integrated with an inverted microscope. mRNA from the aspirate was then selectively captured using magnetic nanoparticles from NVIGEN. The single-cell RNAseq data from the 16 individual cells within the tissue slice provided high alignment rates (80%) with control tissue RNA extracts. The controls were not processed using the NVIGEN nanoparticle and hybridization buffers and included six samples of nanopipette aspirates from individual cells within an intact tissue and two samples from cultured cells. In addition to providing high-quality samples for sequencing, this process proved to be exceptionally simple, quick, and efficient as the entire cell targeting and aspiration process is fully automated.

## Materials & methods

### Nanopipettes & SICM setup

Nanopipettes with a mean diameter of 30 (± 10) nm were fabricated from quartz capillaries using the laser puller P-2000 manufactured by Sutter Instrument, Novato, CA, and were back filled with 1 X PBS 7.4. An Ag/AgCl wire was inserted into the barrel of the nanopipette, while a second Ag/AgCl wire was as reference/ counter electrode. The wires and the nanopipettes are integrated to the SU10 nanopipette controller unit supplied by Yokogawa Electric. The SU10 controller unit with its proprietary software enables fully automated control of the nanopipette in approaching and penetrating a single-cell within a tissue slice.

### Single-cell RNAseq library preparation

Nanopipette aspirates of sub-single-cell content were directly transferred into 8 ul each of NVIGEN single-cell mRNA capture cocktail and processed using the NVIGEN mRNA capture kit (Cat# K81004) containing biotinylated mRNA capture oligos, and capture and hybridization buffers. After overnight incubation under vortexing, 70 μl of NVIGEN mRNA capture magnetic bead-streptavidin conjugates (Cat# 61002 and 21005C) were washed in beads washing buffer and redispersed in 35 μl of elution buffer, 1 μl of which was then added to each RNA solution and incubated with the samples under vortexing for 1.5 hours, then captured single-cell mRNA were magnetically separated from other contents and dispersed into 1 μl of elution buffer. Subsequently, NVIGEN reverse transcription (RT) cocktail was applied to each sample with 0.5 μl each of NVIGEN oligo-dT, random priming, and template switching oligos to enable whole transcriptome sequencing. Single-cell barcodes are designed into the NVIGEN oligos used in these experiments to enable high throughput multiplexing of large number of single-cell RNAseq sample preparation. 1.5 μl of NVIGEN sc-RNAseq RT enhancer buffer (Cat# K81005-Enhance Buffer) was also incorporated in the RT cocktail to enhance the RT yield. After cDNA synthesis, each sample went through NGS library preparation with the NVIGEN single-cell RNAseq library kit (Cat# K81005). Briefly, cDNA was amplified by PCR with specifically designed primers, followed by the PCR cleanup with NVIGEN DNA sizing cleanup Beads (Cat# K61001-Easy) at 0.5X beads vs. sample ratio. Afterwards, a second PCR reaction was performed using NVIGEN NGS sequencing primers followed by NVIGEN DNA sizing beads (Cat# K61001-Easy) cleanup at 0.77 beads vs. sample volume ratio. The eluates went through further PCR amplification with Illumina sequencing dual index primers for sample barcoding. After the final PCR reaction, samples were cleaned-up with NVIGEN DNA sizing cleanup beads (Cat# K61001-Easy) at 1.0X beads vs. sample volume ratio. Final RNAseq libraries were eluted into the elution buffer with library yields quantified using Picogreen (Thermo Fisher Scientific, CA) optical method. Sc-RNAseq library profile was characterized using a Bioanalyzer (Agilent). For sequencing, all single-cell RNAseq libraries were pooled together, followed by a cleanup and enrichment step using NVIGEN DNA sizing cleanup beads (Cat# K61001-Easy) at a 1.0X beads vs. RNAseq library sample ratio. The pooled and cleaned-up library was characterized using Bioanalyzer before loading to a sequencer. All buffers, oligos and magnetic beads for the single-cell RNAseq library preparation were obtained from NVIGEN. (www.nvigen.com).

### Single-cell sequencing library analysis

The sample sequencing reads were processed with Trimmomatic to trim sequencing adapters then mapped with STAR. Mapped reads were counted with HTseq-count to get GeneCounts and annotated with the GencodeV37 gene_type annotations. Sample sequencing FASTQ were processed with Trimmomatic-0.36 to trim sequencing adapters then mapped to hg38 assembly of the human genome with STAR 2.7.3a. Mapped reads were counted with HTseq-count to get GeneCounts and annotated with the GencodeV37 gene type annotations. Comparative plots and correlation statistics were generated with seaborn and ggplot in Python-3.7 and R-3.3.3, respectively.

## Results and discussion

**Fig 1:**
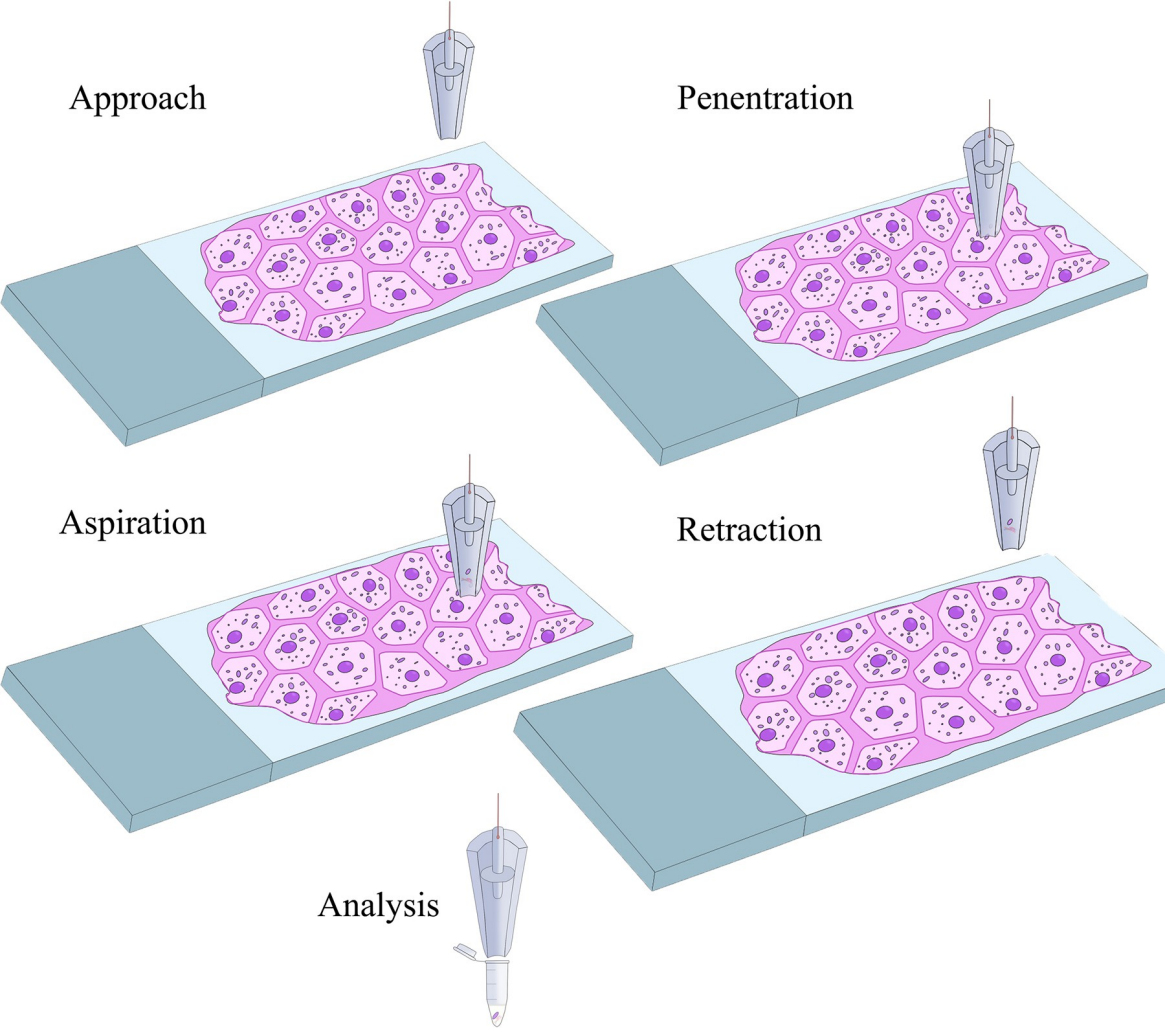
Schematics of the automated single-cell nanobiopsy. The schematics depicts the approach, penetration into the cytoplasm, followed by controlled aspiration of cytoplasmic material of a single cell in an intact tissue slice, retraction of the nanopipette, and delivery of the aspirated material into the PCR tube. Scheme is not to scale.

**Fig 2:**
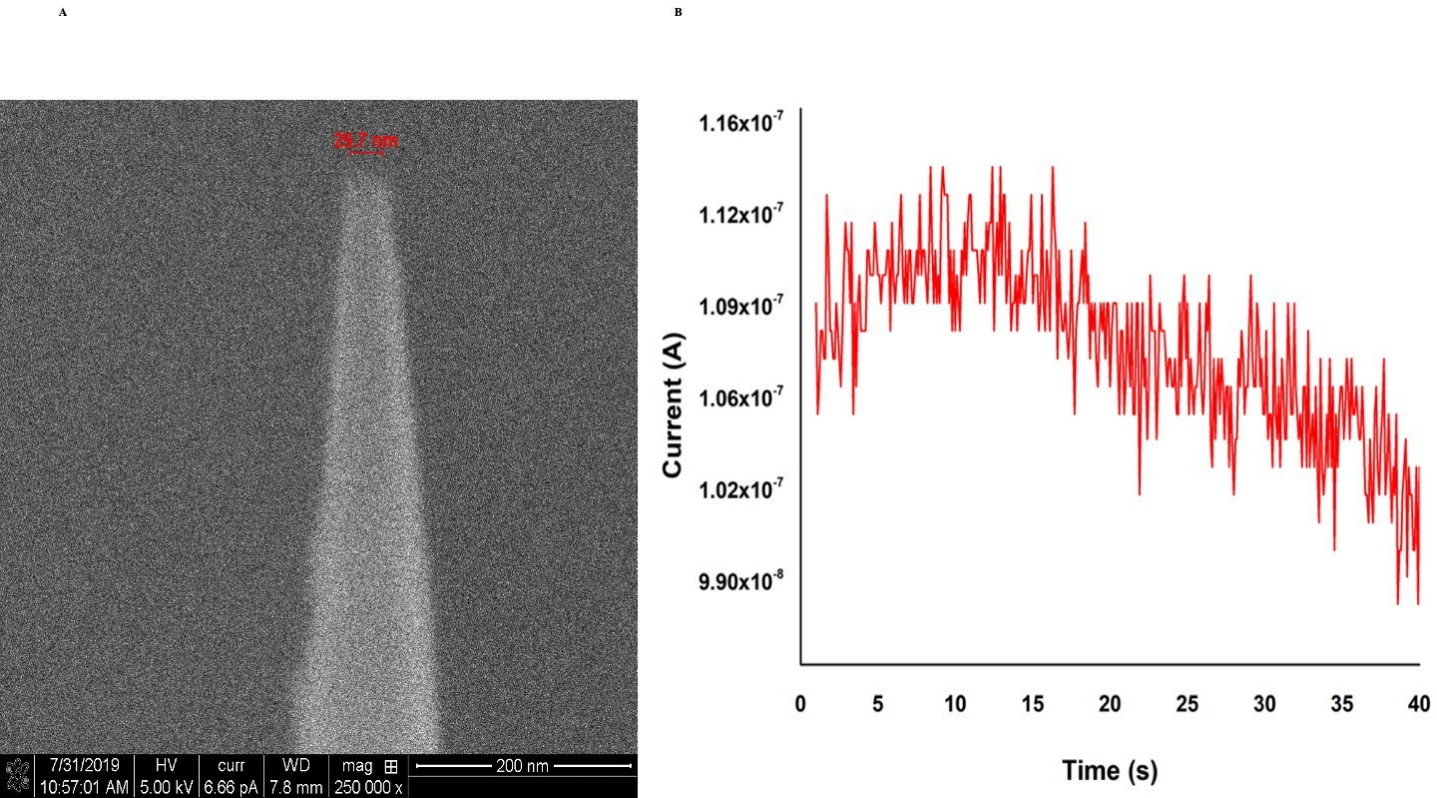
SEM image and Current Vs time plot. SEM image of the nanopipette tip with a pore diameter of ~30 nm (A) and typical, current Vs time plot observed during aspiration of the intracellular matrix using -500 mV for 40 s (B).

The integration of nanopipette with the SICM was achieved as mentioned in our previous publication [8] barring a few modifications. Briefly, nanopipettes with a pore diameter of 30 (±10) nm were backfilled with 1X PBS, pH 7.2 and an Ag/AgCl electrode was inserted into the nanopipette. When the tip of the nanopipette is in contact with an aqueous solution, the application of negative potential will cause a change in surface tension resulting in the movement of the aqueous solution into the nanopipette. Since the tissue samples are usually non-aqueous, the tissue sample slides were placed in petri dishes, and 1 mL of 1X PBS (pH 7.2) was spread onto the slide surfaces, which was sufficient to create a thin liquid layer over the tissue slice. When the nanopipette is lowered to interface with the aqueous layer on top of the tissue sample, a positive bias is applied. This creates an ionic flow between the liquid-liquid interface that acts as a feedback loop to monitor the position of the nanopipette. The custom-designed software provided by Yokogawa Electric directs the nanopipette tip into the cell cytoplasm. The controlled aspiration of the cell cytoplasm is achieved by applying a bias potential of -500 mV DC for 40 s once the nanopipette pierces the cell membrane. A graphical representation of the nanopipette-based aspiration process is shown in Fig 1. At the end of the 40s, the potential is withdrawn, and the electrical circuit moves to an open state, followed by pipette withdrawal. As shown in Fig 2A, nanopipettes with a pore diameter of 30 nm were used for our experiments. Fig 2B represents the current response observed during the 40s of aspiration. The aspirated content is then transferred into a 5μL droplet of the cocktail and kept at 4°C till further processing. Commercially purchased tissue slices of a thickness of 20 um were successfully fixed onto a molecularly modified glass slide. As a proof of concept, intracellular aspiration was carried out on 22 individual cells from within the tissue, along with controls. Since the nanopipette aspiration protocol has been modified compared to the one published by *Actis et*. *al*. [8], aspiration was also carried out from two cells maintained in cultured media. The comparison of the current response between single-cell aspirations in a tissue sample versus cells cultured in a petri dish was identical proving that the aspiration was successful from single-cells within the tissue. The use of automated nanopipettes for aspirating intracellular components from single cells within a tissue is highly advantageous over current methods as it ensures that the aspirate is from a single cell and the process is quick.

**Fig 3:**
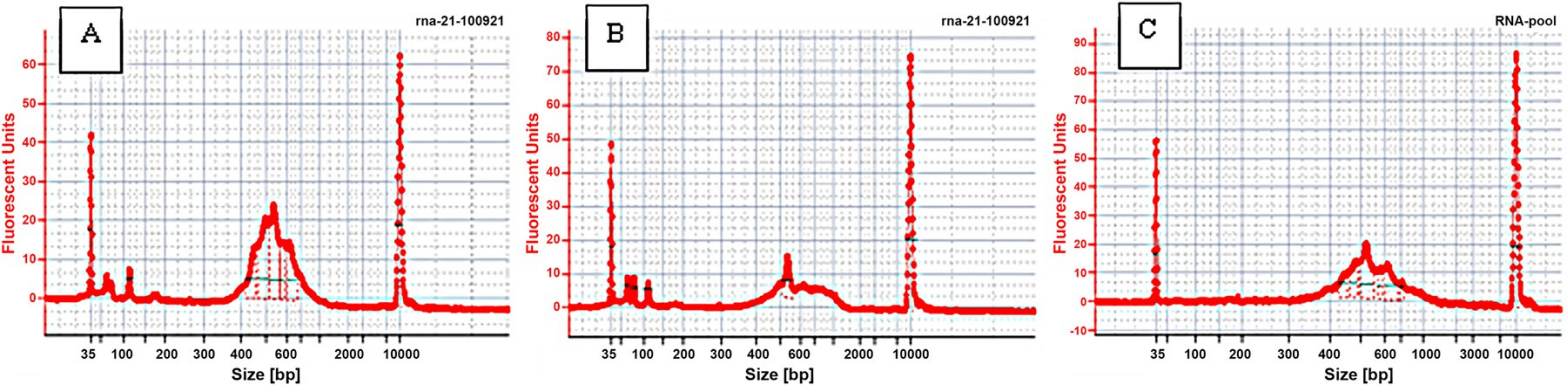
Bioanalyzer profiles. Bioanalyzer profiles of RNAseq libraries from as-made single-cell RNAseq libraries without purifying primers, adaptors, and dimers (Fig 3A & 3B). Fig 3C shows purified and pooled samples prior to cDNA sequencing.

**Fig 4:**
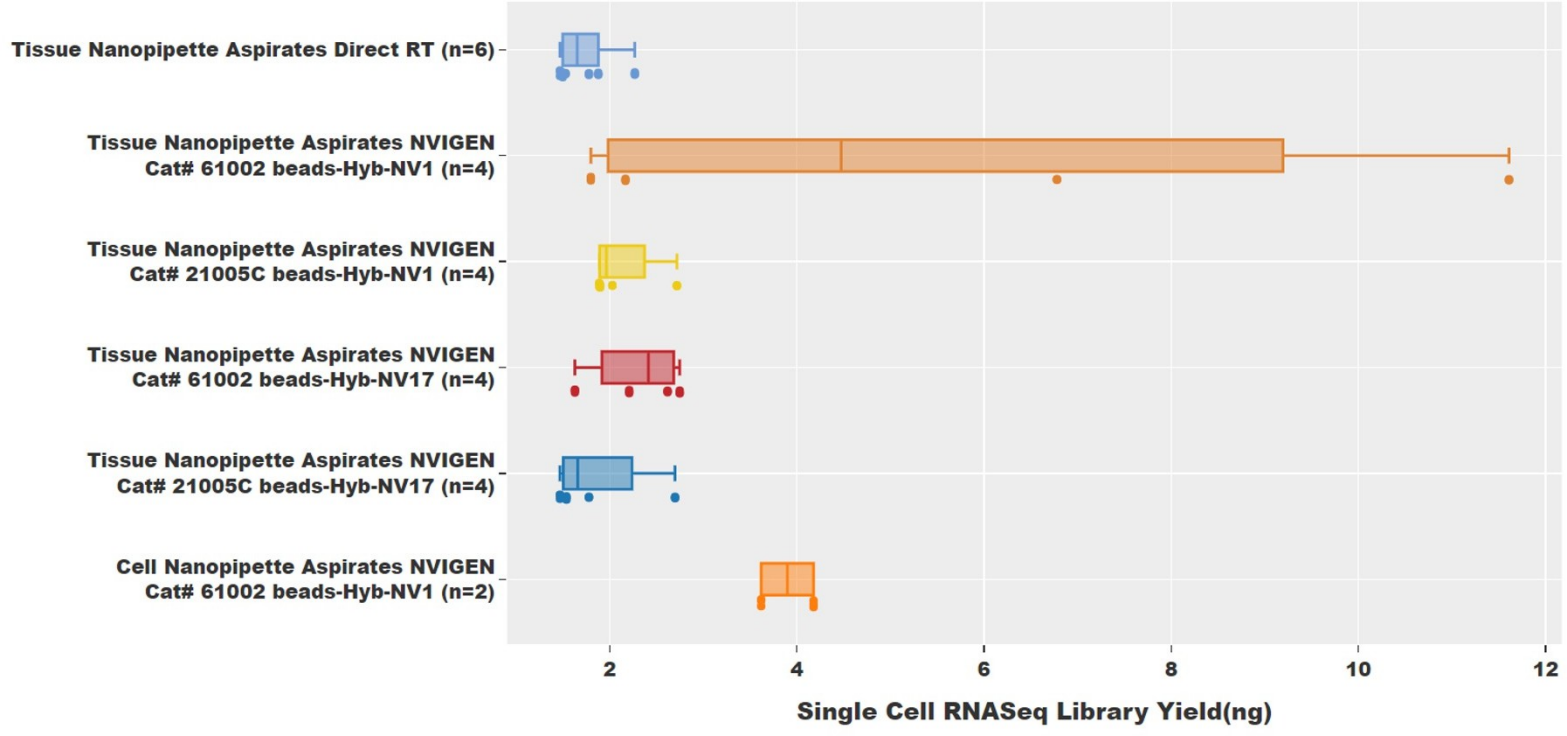
Comparison of RNAseq library yield. Higher library yields were obtained when different NVIGEN magnetic nanoparticles are used for cDNA capture.

The sub-cellular aspirates mixed with the NVIGEN mRNA capture cocktail were pooled and cleaned up before characterization using a bioanalyzer and sequencing. In total, 16 nanopipette aspirate samples were processed with the NVIGEN nanoparticle and hybridization buffer. All the samples were successfully processed through NVIGEN’s highly sensitive RNAseq sample and assay workflow to generate NGS data. The bioanalyzer profiles of RNAseq libraries from 2 single-cell nanopipette aspirates within the tissue showed the library peak centered slightly over 500bp (Fig 3A & 3B). From the data, it was also observed that the as-made single-cell RNAseq libraries still contained primers, adaptors, and dimers. Hence the 16 samples of RNAseq libraries were pooled together and cleaned using NVIGEN Next Generation Sequencing (NGS) library clean-up beads (Cat# K61001) to remove the primers, adaptors, and their dimers. As shown in Fig 3C, the bioanalyzer profile of the pooled library exhibits a clean peak with no traces of adaptors, primers, or dimers. Experiments were conducted to understand the efficiency of the different NVIGEN nanoparticles and hybridization buffers toward library yield preparation. Fig 4 shows the RNAseq library yield for each of the 16 samples in groups designed to compare the sample preparation conditions. As seen from Fig 4, NVIGEN magnetic nanoparticles with Cat# 61002 provided higher RNAseq library yield than NVIGEN nanoparticles with Cat# 21005C while using both hybridization buffers NV-1 and NV-17. Also, experiments conducted with hybridization buffer NV1 provided higher RNAseq library yield than Hyb-NV17 buffer while using both magnetic nanoparticles. The library yield for the Cat# 61002 was observed to be 5.59 ng and 2.30 ng while using the Hyb-NV1 and Hyb-NV17, respectively, while Cat# 21005C with Hyb-NV1 and Hyb-NV17 showed a yield of 2.14 ng and 1.87 ng, respectively. The data shows that the single-cell nanopipette aspirate RNA samples from intact tissue processed using NVIGEN magnetic nanoparticle mRNA capture resulted in higher library yield than nanopipette aspirate tissue RNA samples processed without nanoparticle capture. The nanopipette aspirate RNA samples from single-cell cultured cells were also successfully processed with NVIGEN Cat# 61002 nanoparticle capture and Hyb-NV1 buffer with an average library yield of 3.9 ng.

**Fig 5:**
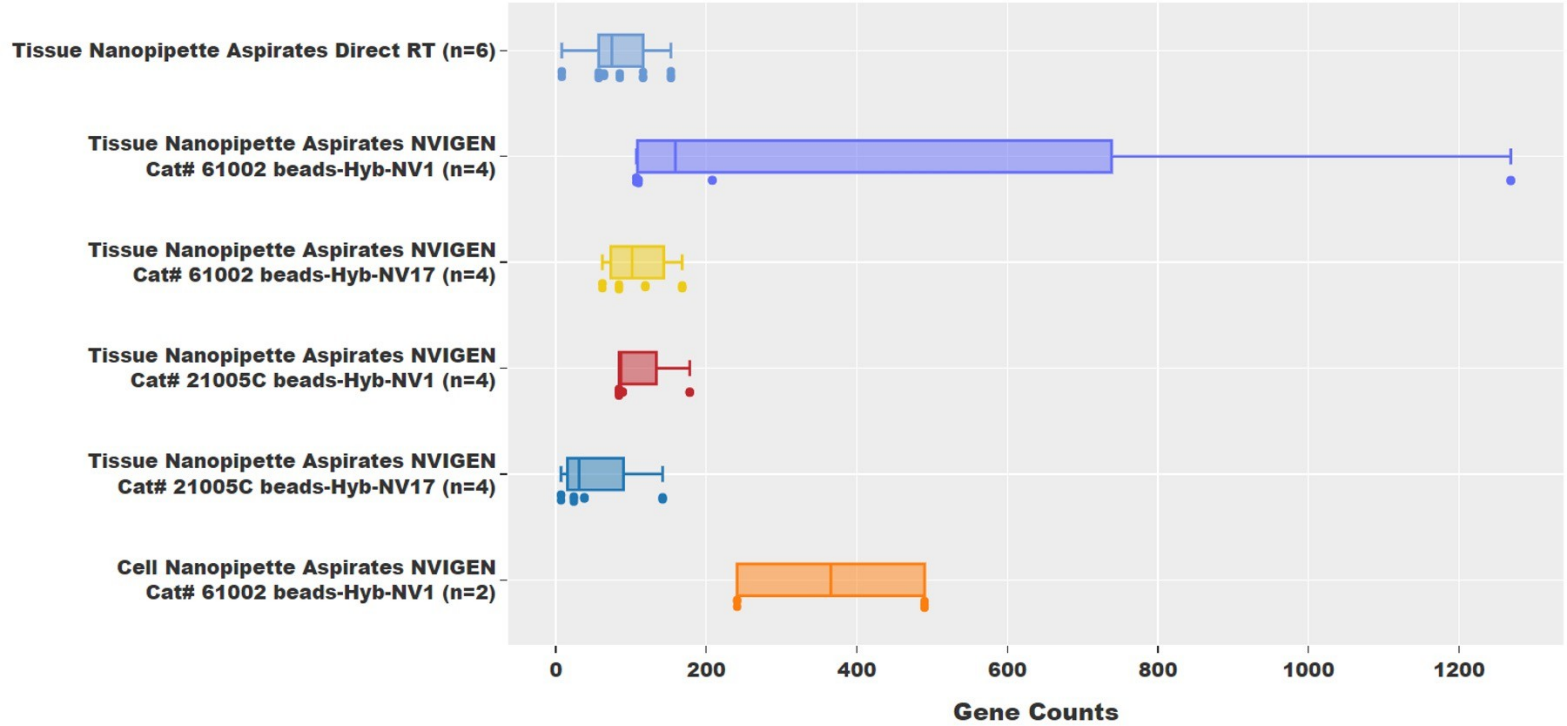
Gene counts plot. The gene count plots revealed hundreds of genes were isolated from each of the aspirated samples.

RNAseq data from all 24 samples were analyzed and are shown in Fig 5 through Fig 9. Gene counts from each sample were also analyzed and plotted as shown in Fig 5. It was observed that single-cell RNA nanopipette aspirate samples processed with the NVIGEN nanoparticles and hybridization buffer had better gene counts with the best gene counts per sample totaling 1269 genes being identified. While the average gene count of the single-cell RNA nanopipette aspirate samples directly processed without nanoparticle capture is 81 genes from 30477 sequencing reads with normalized gene counts per million reads of 4245. On average, the gene counts for the 16 samples from tissue single-cell RNA nanopipette aspirate samples processed with nanoparticles were observed to be 154 genes per aspiration. Of these, an average gene count of 424 with sequencing reads of 271968 per cell was obtained using the NVIGEN Cat# 61002 beads and Hyb-NV1 buffer for mRNA capture. This corresponds to 1494 gene counts per million reads. A nanopipette single-cell tissue aspiration had the highest gene count of 28950 per million reads using the NVIGEN Cat# 21005C nanoparticles and Hyb-NV17 buffer with 24 gene counts from 829 sequencing reads. The NVIGEN Cat# 61002 beads and Hyb-NV17 provided an average of 1683 gene counts per million reads with an average of 60 gene counts from 63874 sequencing reads per sample. The NVIGEN Cat# 21005C beads and Hyb-NV1 provided an average of 2339 gene counts per million reads with an average of 109 gene counts from 48364 sequencing reads per sample.

**Fig 6:**
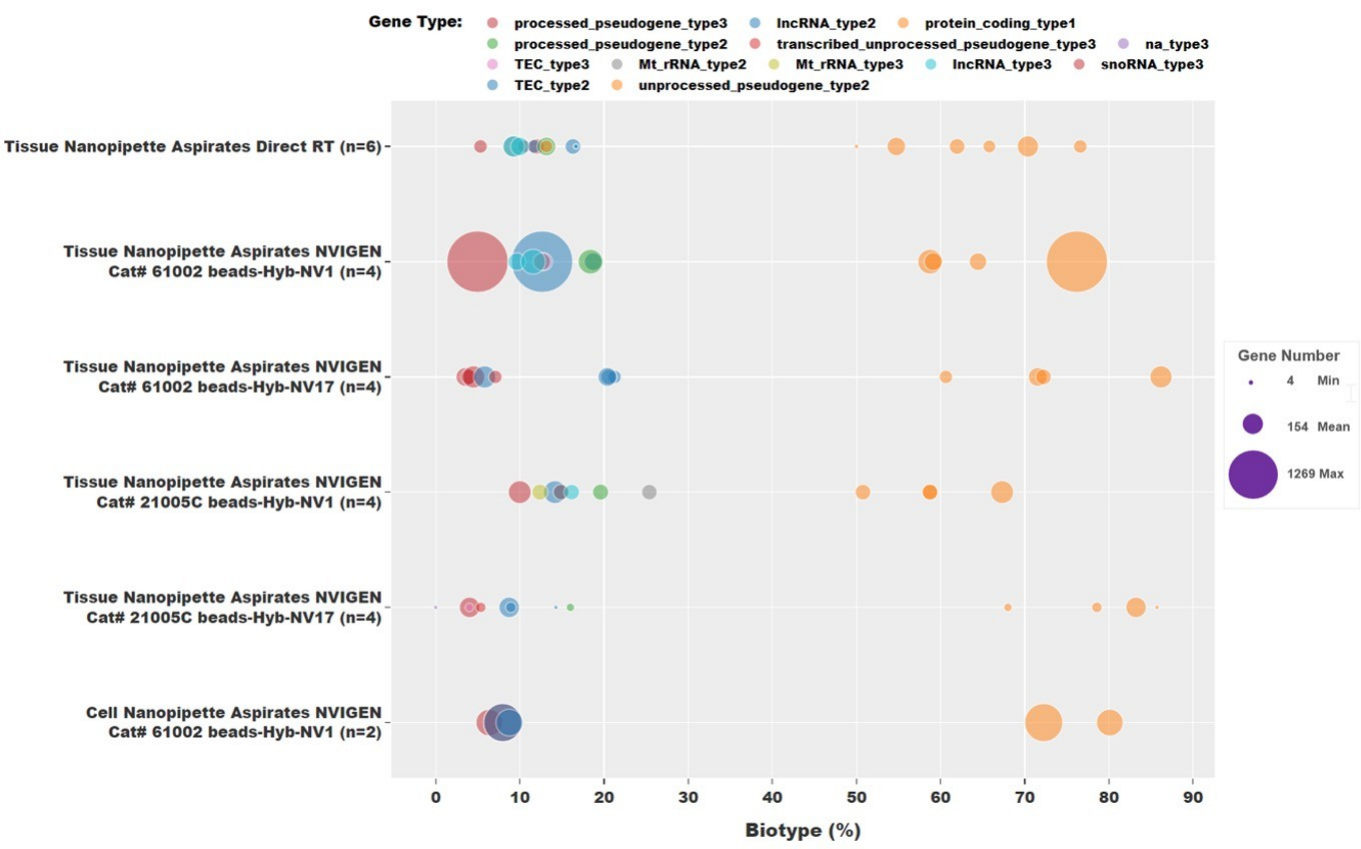
RNAseq data. Analysis of RNAseq data revealed that majority of reads aligned to the protein coding region with the dot size representing the gene count within each biotype.

Similarly, for single-cell RNA nanopipette aspirate samples from cultured cells and processed with NVIGEN mRNA capture nanoparticles, the gene counts per million reads stood at 3400, and 3321, with respective gene counts of 490 and 241 from 144090 and 72568 reads, respectively. Fig 6 represents the data for alignment of RNAseq reads on all biotypes using the dot size to represent the number of gene counts within each biotype for each of the 24 samples. From the plot, it is evident that most of the sequencing reads for each sample aligned to the protein coding region. A high correlation of about 80% could be achieved for each sample group of the single-cell RNA nanopipette aspirate samples.

**Fig 7:**
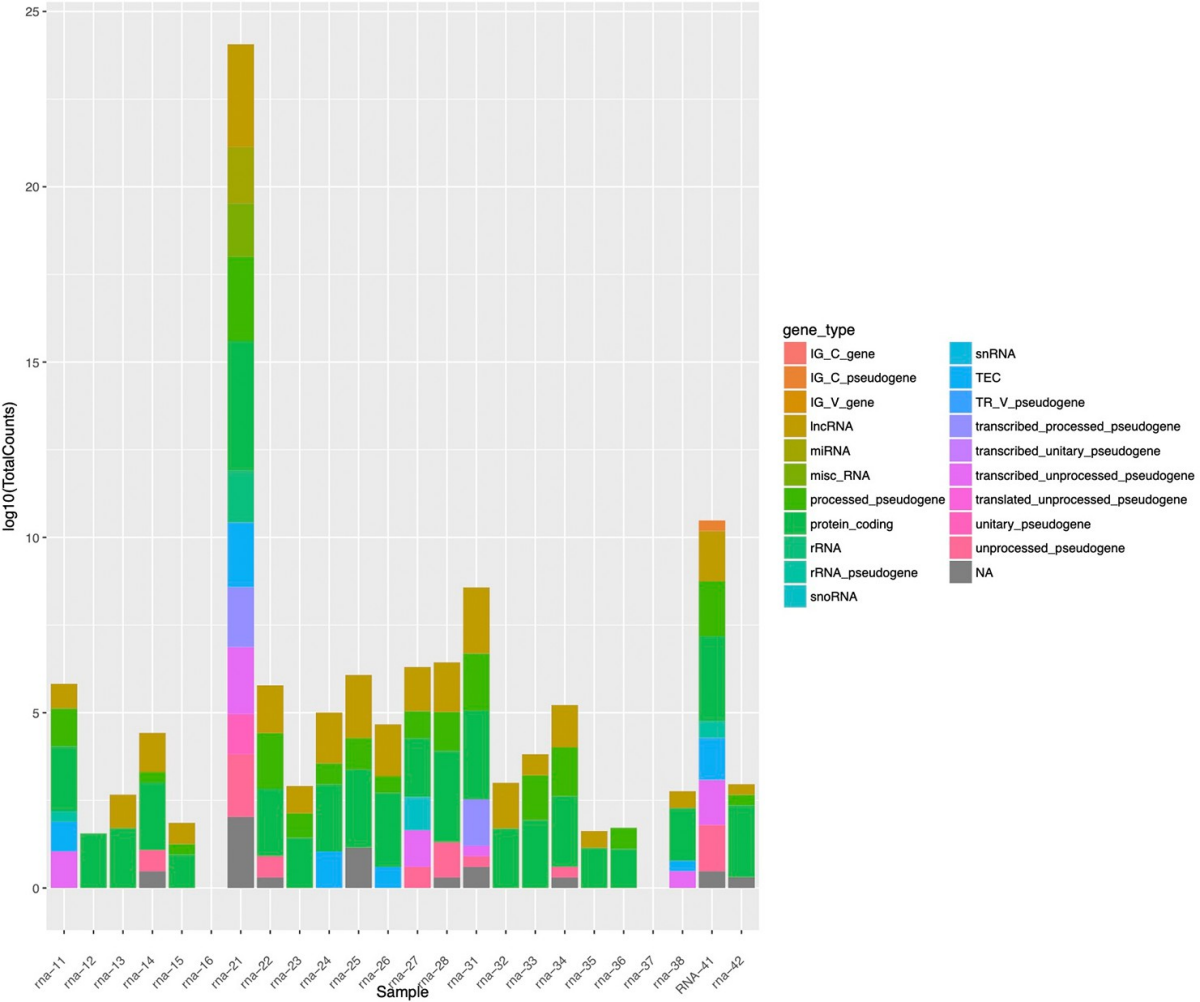
Stacked bar plot. Stranded GeneCounts from mapping were log10 transformed and plotted as a stacked bar plot, indicating proportions of gene annotations for the mapped reads.

**Fig 8:**
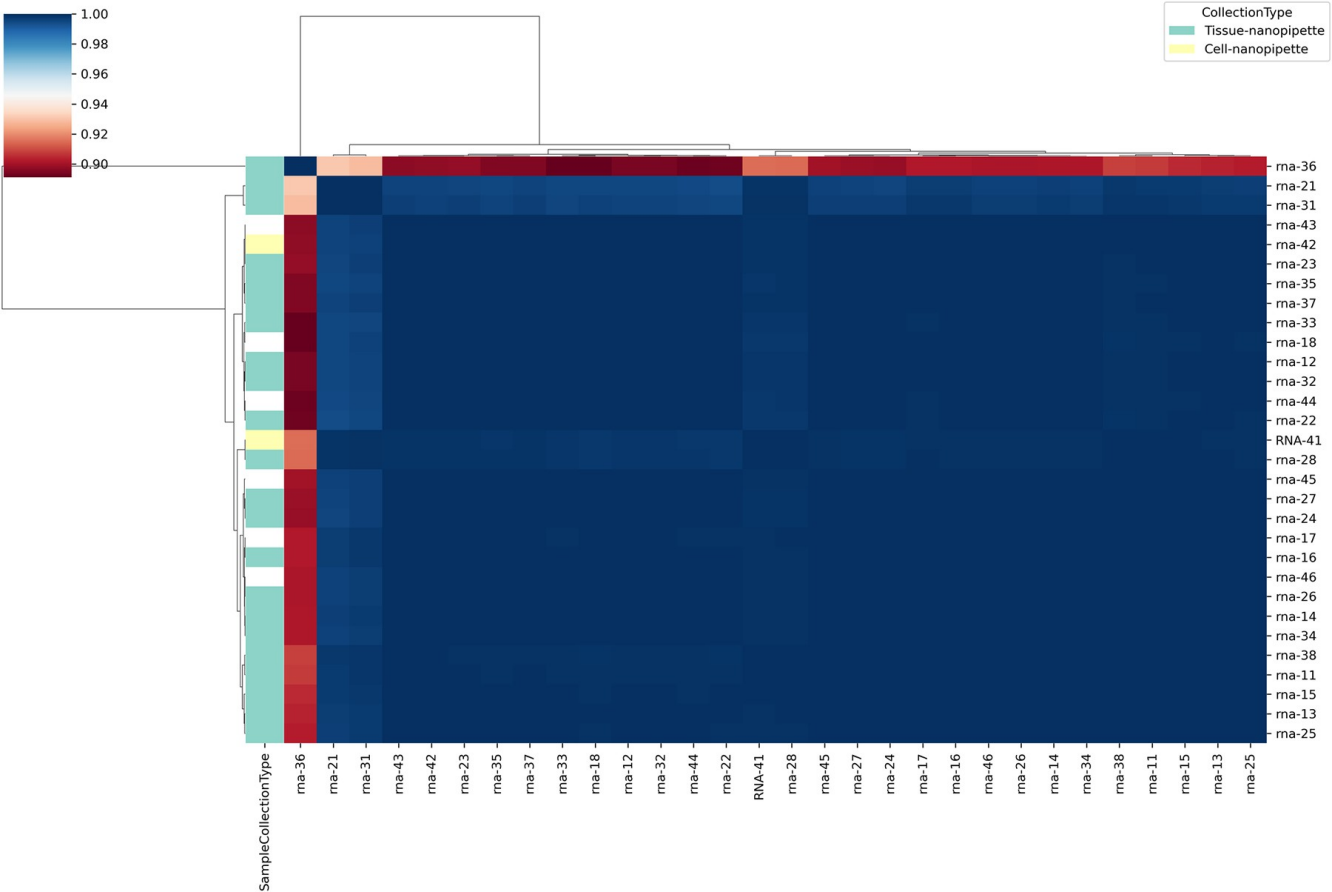
Clustered pairwise correlation matrix. Pairwise correlations between the Stranded GeneCounts from mapped reads were calculated then clustered in pairwise correlation matrix. The pairwise correlation scores and clustering demonstrates high correlation in detected genes between the various aspirations.

**Fig 9:**
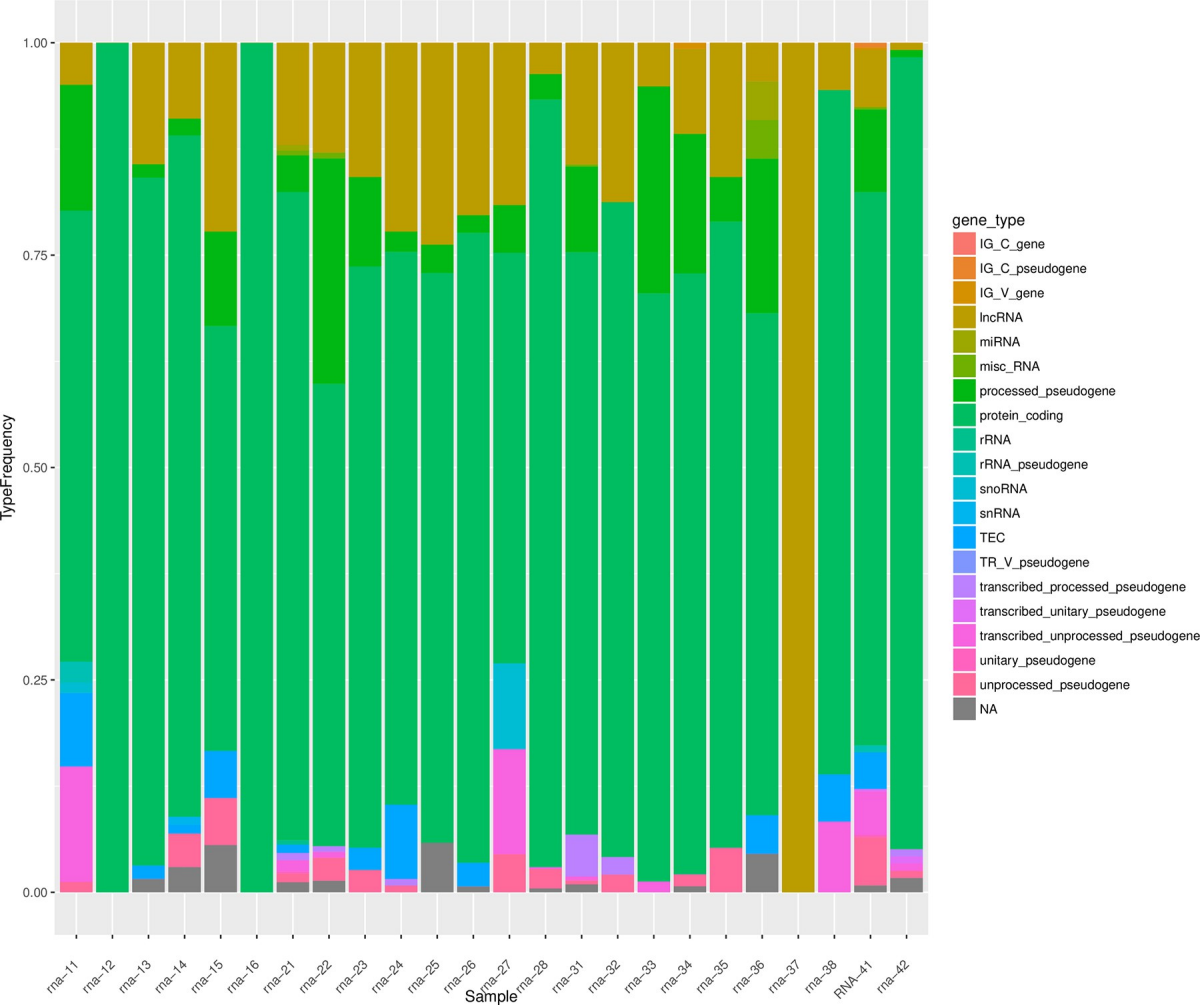
Frequency bar plot. Frequency bar plot representing un-stranded and 1^st^ strand mapping, showing mapping results were primarily correlated with the 1^st^ strand mapping.

The GeneCounts for each sample were log10 transformed and plotted as a stacked bar plot and color based on the proportion associated with each detected Gencode RNA annotation as shown in Fig 7. Based on the proportions of the colors associated with each gene type annotation, it is evident that RNAs isolates from nanopipette aspiration samples compared to bulk tissue or cell samples are highly correlated Fig 8. This is further confirmed with the frequency bar plots (Fig 9) demonstrating the bulk of the detected genes in each of the samples to be protein coding genes, except for sample rna-37 (a nanopipette aspiration on tissue) which has more of a proportion of lncRNA genes detected. These results have conclusively proved that combining the nanopipette technology with the NIVGEN magnetic nanoparticles has created a powerful tool that allows for simple, efficient, and high throughput isolation of quality mRNA from individual cells in an intact tissue population.

## Conclusion

We successfully demonstrated RNA isolation for the first time (to our knowledge) and obtained high-quality sequencing reads from single cells in intact sliced tissue by combining the nanopipette technology with the magnetic nanoparticles from NVIGEN. Experimental data have proven that RNA obtained from nanobiopsies, bulk tissue, and cell samples are highly correlated, which is evidence of the effectiveness of the technology for obtaining high-quality reads without contamination. By pairing the nanopipette technology with the highly sensitive magnetic nanoparticle-enabled RNA capture and sequencing library preparation workflow, we have achieved single-cell RNAseq with only 1–5% of sub-single-cell RNA content obtained from intact fresh frozen tissue samples. With the proposed technology, the single-cell is still viable for subsequent sampling and sequencing to get longitudinal information. This technology provides a dramatic advantage compared to most other single-cell RNAseq approaches that consume the entire intracellular content. This allows for new application opportunities, including measurement of single-cell transcriptome data sequentially pre- and post-drug treatment and continuous monitoring of therapeutic responses. By integrating the nanopipettes with the SU10 controller unit from Yokogawa Electric, the technology is fully automated and capable of high throughput sampling. This scientific advancement helps overcome the challenges observed in other commercially available technologies involving physical dissociation and isolation of individual cells from a tissue sample and amplifying the genome of that complete single cell to acquire sufficient material for down- stream analyses. With the worldwide commercial availability of the NVIGEN magnetic nanoparticle kit and the Yokogawa Electric SU10 controller, this new technology is poised to rapidly advance our understanding of genomic variability within tissues as it reaches the hands of the end user.

## References

[1] Turner W. The cell theory, past and present. Journal of anatomy and physiology. 1890;24(Pt 2):253. 17231856 PMC1328050

[2] Gawad C, Koh W, Quake SR. Single-cell genome sequencing: current state of the science. Nature Reviews Genetics. 2016;17(3):175–88. doi: 10.1038/nrg.2015.16 26806412

[3] Emmert-Buck MR, Bonner RF, Smith PD, Chuaqui RF, Zhuang Z, Goldstein SR, et al. Laser capture microdissection. Science. 1996;274(5289):998–1001. doi: 10.1126/science.274.5289.998 8875945

[4] Navin NE. Cancer genomics: one cell at a time. Genome biology. 2014;15(8):1–13. 10.1186/s13059-014-0452-9 PMC4281948 25222669

[5] Laforge FO, Carpino J, Rotenberg SA, Mirkin MV. Electrochemical attosyringe. Proceedings of the National Academy of Sciences. 2007;104(29):11895–900. doi: 10.1073/pnas.0705102104 17620612 PMC1924598

[6] Rodolfa KT, Bruckbauer A, Zhou D, Korchev YE, Klenerman D. Two‐component graded deposition of biomolecules with a double‐barreled nanopipette. Angewandte Chemie. 2005;117(42):7014–9. doi: 10.1002/anie.200502338 16249993

[7] Seger RA, Actis P, Penfold C, Maalouf M, Vilozny B, Pourmand N. Voltage controlled nano-injection system for single-cell surgery. Nanoscale. 2012;4(19):5843–6. doi: 10.1039/c2nr31700a 22899383 PMC4406976

[8] Actis P, Maalouf MM, Kim HJ, Lohith A, Vilozny B, Seger RA, et al. Compartmental genomics in living cells revealed by single-cell nanobiopsy. ACS nano. 2014;8(1):546–53. doi: 10.1021/nn405097u 24279711 PMC3946819

[9] Nashimoto Y, Takahashi Y, Zhou Y, Ito H, Ida H, Ino K, et al. Evaluation of mRNA localization using double barrel scanning ion conductance microscopy. ACS nano. 2016;10(7):6915–22. doi: 10.1021/acsnano.6b02753 27399804

[10] Umehara S, Pourmand N, Webb CD, Davis RW, Yasuda K, Karhanek M. Current rectification with poly-l-lysine-coated quartz nanopipettes. Nano letters. 2006;6(11):2486–92. doi: 10.1021/nl061681k 17090078 PMC2948113

[11] Actis P, Mak AC, Pourmand N. Functionalized nanopipettes: toward label-free, single cell biosensors. Bioanalytical reviews. 2010;1(2):177–85. doi: 10.1007/s12566-010-0013-y 20730113 PMC2918800

[12] Ding S, Gao C, Gu L-Q. Capturing single molecules of immunoglobulin and ricin with an aptamer-encoded glass nanopore. Analytical chemistry. 2009;81(16):6649–55. doi: 10.1021/ac9006705 19627120 PMC3009471

[13] Gao R, Lin Y, Ying Y-L, Hu Y-X, Xu S-W, Ruan L-Q, et al. Wireless nanopore electrodes for analysis of single entities. Nature Protocols. 2019;14(7):2015–35. doi: 10.1038/s41596-019-0171-5 31168087

[14] Liu G-C, Gao M-J, Chen W, Hu X-Y, Song L-B, Liu B, et al. pH-modulated ion-current rectification in a cysteine-functionalized glass nanopipette. Electrochemistry Communications. 2018;97:6–10.

[15] Keating SM, Taylor DL, Plant AL, Litwack ED, Kuhn P, Greenspan EJ, et al. Opportunities and challenges in implementation of multiparameter single cell analysis platforms for clinical translation. Clinical and Translational Science. 2018;11(3):267–76. doi: 10.1111/cts.12536 29498218 PMC5944591

[16] Femino AM, Fay FS, Fogarty K, Singer RH. Visualization of single RNA transcripts in situ. Science. 1998;280(5363):585–90. doi: 10.1126/science.280.5363.585 9554849

[17] Raj A, Peskin CS, Tranchina D, Vargas DY, Tyagi S. Stochastic mRNA synthesis in mammalian cells. PLoS biology. 2006;4(10):e309. doi: 10.1371/journal.pbio.0040309 17048983 PMC1563489

[18] Betzig E, Patterson GH, Sougrat R, Lindwasser OW, Olenych S, Bonifacino JS, et al. Imaging intracellular fluorescent proteins at nanometer resolution. science. 2006;313(5793):1642–5. doi: 10.1126/science.1127344 16902090

[19] Rust MJ, Bates M, Zhuang X. Sub-diffraction-limit imaging by stochastic optical reconstruction microscopy (STORM). Nature methods. 2006;3(10):793–6. doi: 10.1038/nmeth929 16896339 PMC2700296

[20] Lubeck E, Coskun AF, Zhiyentayev T, Ahmad M, Cai L. Single-cell in situ RNA profiling by sequential hybridization. Nature methods. 2014;11(4):360–1. doi: 10.1038/nmeth.2892 24681720 PMC4085791

[21] Shah S, Lubeck E, Schwarzkopf M, He T-F, Greenbaum A, Sohn CH, et al. Single-molecule RNA detection at depth by hybridization chain reaction and tissue hydrogel embedding and clearing. Development. 2016;143(15):2862–7. doi: 10.1242/dev.138560 27342713 PMC5004914

[22] Nesvet JC, Antilla KA, Pancirer DS, Lozano AX, Preiss JS, Ma W, et al. Giant Magnetoresistive Nanosensor Analysis of Circulating Tumor DNA Epidermal Growth Factor Receptor Mutations for Diagnosis and Therapy Response Monitoring. Clinical Chemistry. 2021;67(3):534–42. doi: 10.1093/clinchem/hvaa307 33393992 PMC12118574

[23] Chaves G, Stanley J, Pourmand N. Mutant huntingtin affects diabetes and Alzheimer’s markers in human and cell models of Huntington’s disease. Cells. 2019;8(9):962. doi: 10.3390/cells8090962 31450785 PMC6769852

[24] Ho MCW. Discovery of active cis-regulatory elements and transcription factor footprints in nematodes using functional genomics approaches: California Institute of Technology; 2015.

[25] Grudzien-Nogalska E, Wu Y, Jiao X, Cui H, Mateyak MK, Hart RP, et al. Structural and mechanistic basis of mammalian Nudt12 RNA deNADding. Nature chemical biology. 2019;15(6):575–82. doi: 10.1038/s41589-019-0293-7 31101919 PMC6527130

[26] Grudzien-Nogalska E, Wu Y, Jiao X, Cui H, Hart RP, Tong L, et al. Structural and biochemical studies define Nudt12 as a new class of RNA deNADding enzyme in mammalian cells. bioRxiv. 2018:474478.

[27] Sallé J, Xie J, Ershov D, Lacassin M, Dmitrieff S, Minc N. Asymmetric division through a reduction of microtubule centering forces. Journal of Cell Biology. 2019;218(3):771–82. doi: 10.1083/jcb.201807102 30563876 PMC6400563

[28] Jin J, Niu X, Zou L, Li L, Li S, Han J, et al. AFP mRNA level in enriched circulating tumor cells from hepatocellular carcinoma patient blood samples is a pivotal predictive marker for metastasis. Cancer Letters. 2016;378(1):33–7. doi: 10.1016/j.canlet.2016.04.033 27160647

[29] Zou L, Su W, Wang M, Huang W, Zhao H, Zhang E, et al. Characterization of a functional recombinant human creatine kinase-MB isoenzyme prepared by tandem affinity purification from Escherichia coli. Applied Microbiology and Biotechnology. 2017;101(14):5639–44. doi: 10.1007/s00253-017-8286-5 28432439

[30] Tang CK, Vaze A, Shen M, Rusling JF. High-throughput electrochemical microfluidic immunoarray for multiplexed detection of cancer biomarker proteins. ACS sensors. 2016;1(8):1036–43. doi: 10.1021/acssensors.6b00256 27747294 PMC5058420

[31] Aguero T, Jin Z, Owens D, Malhotra A, Newman K, Yang J, et al. Combined functions of two RRMs in Dead‐end1 mimic helicase activity to promote nanos1 translation in the germline. Molecular reproduction and development. 2018;85(12):896–908. doi: 10.1002/mrd.23062 30230100 PMC6294668

[32] Gong Q, Long Z, Zhong FL, Teo DET, Jin Y, Yin Z, et al. Structural basis of RIP2 activation and signaling. Nature communications. 2018;9(1):1–13. 10.1038/s41467-018-07447-9 PMC6255760 30478312

[33] Arokiaraj M, Menesson E, Feltin N. Magnetic iodixanol–a novel contrast agent and its early characterization. JMV-Journal de Médecine Vasculaire. 2018;43(1):10–9. 10.1016/j.jdmv.2017.11.002 29425536

[34] Fu A, Wilson RJ, Smith BR, Mullenix J, Earhart C, Akin D, et al. Fluorescent magnetic nanoparticles for magnetically enhanced cancer imaging and targeting in living subjects. ACS nano. 2012;6(8):6862–9. doi: 10.1021/nn301670a 22857784 PMC3601027

